# Effects of brimonidine tartrate 0.2 and 0.15% ophthalmic solution on the static and dynamic pupil characteristics

**DOI:** 10.3389/fmed.2023.1160414

**Published:** 2023-05-15

**Authors:** Jing Yang, Xiaodi Zhang, Mei Zhong, Yanhui Bai, Wentao Liu, Jinge Hu, Weiqun Wang

**Affiliations:** Department of Ophthalmology, The First Affiliated Hospital of Zhengzhou University, Henan Eye Hospital, Zhengzhou, China

**Keywords:** ICL V4c, high myopia, brimonidine tartrate, visual quality, scotopic pupil

## Abstract

**Aims:**

To investigate the differences between 0.2 and 0.15% brimonidine tartrate eye drops for anti-mydriatic effects and the optical quality under different light conditions.

**Methods:**

This prospective study involved 80 consecutive high myopia patients undergoing implantation of a V4c ICL. The patients were randomly instilled with brimonidine 0.2 and 0.15% 2 weeks postoperatively. Visual quality, pupil center, pupil size, and refraction under different light conditions were measured before and 0.5 h after brimonidine administration. A symptom questionnaire was also evaluated.

**Results:**

There was no statistical difference in the static and dynamic pupil diameters and velocity after LS between the two groups (*p* > 0.05). The 0.2% group had significant changes in pupil center before and after treatment, while there was no obvious movement of the 0.15% group under all illumination condition (*p* > 0.05). The OSI after treatment of the 0.15% group was lower than that of 0.2% group (*p* = 0.012). The PVA9% and PVA100% of the 0.15% group was higher than that of 0.2% group in the dark (*p* = 0.009, *p* = 0.012). The HOA RMS of the 0.15% group was lower than that of 0.2% group (*p* = 0.016). The QIRC score in the 0.15% group was significantly higher than that in the 0.2% group (*p* = 0.043).

**Conclusion:**

0.15 and 0.2% brimonidine tartrate eye drops had similar anti-mydriatic ability, while 0.15% group had better visual quality than 0.2% concentration, and hardly introduced pupil shift. 0.15% brimonidine tartrate eye drops may be more suitable for patients with nocturnal glare symptoms in the early postoperative period after ICL implantation.

## Introduction

Implantation of phakic intraocular lenses (pIOLs) to correct high myopia can help obtain better vision and visual quality, which is currently a primary surgical method for correcting myopia (1, 2). Currently, the widely used phakic posterior chamber implantable collamer lens is Implantable Collamer Lens (ICL), produced by STAAR Surgical (3–5). Numerous extensive sample size studies have confirmed that it is a safe and predictable operation method (5–8). In the early stage of ICL surgery, some patients complained dysphotopsia at night, such as ring-shaped (9, 10). Recent studies have shown that such visual impairment might be related to pupil dynamics (9–12).

Brimonidine is a selective alpha-2 adrenergic receptor agonist that reduces intraocular pressure by reducing aqueous humor production and increasing uveoscleral outflow, and is a commonly used drug for the treatment of glaucoma and ocular hypertension (13). Brimonidine tartrate eye drops have been reported to be effective in inhibiting pupil dilation (14, 15). It can significantly reduce pupil diameter under scotopic conditions, and can effectively reduce glare and halos after refractive surgery (14, 15). Although previous studies have explored the miotic effect of 0.2 and 0.15% brimonidine tartrate eye drops, no study has compared the differences of effects in anti-mydriatic, on the pupil center, and on the optical quality. The purpose of this study was to objectively and quantitatively compare the effect of 0.2 and 0.15% brimonidine tartrate eye drops on pupil size, pupil center, and visual quality in different light conditions after implantable collamer lens with a central hole implantation.

## Materials and methods

### Study population

A total of 80 eyes of 80 high myopia patients who underwent ICL implantation at the Ophthalmology Department of the First Affiliated Hospital of Zhengzhou University between January 2021 and December 2021 were enrolled in this prospective observational study. Supplementary material S1 shows the baseline data for all patients. All patients received a comprehensive eye examination and were informed of the operation-related risks before surgery. The inclusion criteria were: (1) 20–40 years old; (2) myopia ≥6.00 D, diopter increase ≤0.5 D for 2 consecutive years; (3) anterior chamber depth (ACD) measured by corneal topography measurements (Sirius; Costruzione Strumenti Oftalmici, Florence, Italy) ≥ 2.80 mm, and corneal endothelial cell count ≥2,000/mm^2^; and (4) preoperative intraocular pressure is normal. The exclusion criteria were: (1) previous corneal refractive surgery, internal eye surgery history, or history of eye trauma; (2) combined cataract, glaucoma, amblyopia, retinal detachment, uveitis, incomplete eyelid closure, and other eye diseases; (3) systemic organic diseases that affect recovery from surgery; and (4) psychological and mental diseases. The ethics committee of Zhengzhou University’s First Affiliated Hospital gave its approval to this study, and followed the tenets of the Declaration of Helsinki. Prior to participation in the study, all patients signed a written informed permission form.

### Measurements

Each eye underwent comprehensive preoperative evaluations. The anterior chamber depth (ACD), anterior chamber angle (ACA), central corneal thickness (CCT), and horizontal white-to-white diameter (hWTW) were assessed using Sirius topography (CSO, Florence, Italy). Performed the following measurements and procedures: uncorrected distance visual acuity, corrected distance visual acuity, manifest refraction spherical equivalent (MRSE), intraocular pressure measurement (IOP), endothelial cell density (ECD), and axial length (AL). ICL V4c was calculated based on the vertex formula of the software.^1^ The following parameters were collected 1 month postoperatively: manifest refraction, logMAR UDVA, logMAR CDVA, and IOP.

### Pupillometry

Pupillometry was performed by a single physician under the same environmental conditions 2 weeks postoperatively, 0.2 and 0.15% brimonidine were administered into random eye of two equal groups. Both the patients and the examiner were masked to the ophthalmic solution. All parameters were measured before and 30 min after eye drops administration. To minimize the effect of circadian variation on PD and pupil motility, all measurements were performed at the same time of day (between 9:00 AM and 11:00 AM). Pupillography module of Sirius topography (CSO, Florence, Italy) was used, the measurement shooting page and data display page of pupillography module are shown in Supplementary Figures S1, S2. Static pupillometry was conducted for the PD (mm) under three standardized illumination conditions: scotopic (0.4 lux), mesopic (4 lux), and photopic (40 lux) light conditions. At least three consecutive measurements of PD were taken at each illumination level, and the average values were selected for analysis. After 15 min of dark adaptation, dynamic pupillometry was measured. The capture is begun with the disk rings fully illuminated (500 lux *ca.*), it is switched off at the moment capture begins. In this manner, it is possible to monitor pupil dilation in conditions from photopic to scotopic conditions and analyze pupil size and pupil offset instant by instant. After at least three valid responses were recorded, the average pupil dynamics were automatically quantified, including the initial PD, smallest PD after light stimulation, velocity of pupil dilation within 1 s, within 2 s, and between 1 and 2 s.

### Optical quality measurement

The modulation transfer function (MTF) cutoff frequency, Strehl ratio, objective scattering index (OSI), and predicted visual acuities (PVAs, 100, 20, and 9%) under scotopic and photopic lighting conditions were measured using the Optical Quality Analysis System™ (OQAS; Visiometrics, Terrassa, Spain) preoperatively and at 1 month after surgery. First, scotopic measurements were performed in a dark room, with the addition of black covers on the instrument to rule out any influence of light from the computer screen. Second, photopic measurements were performed after turning on the room light source, and a light reflex was induced by shining a penlight (250 lm) into the contralateral eye. All of the above measurements were performed under the corresponding pupil diameter. All the manifest refractive errors were fully corrected during these measurements. All measurements were performed three times, and the mean value was calculated and recorded. According to the measuring principle of OQAS, participants with lower OSI, higher MTF cutoff, higher Strehl ratio, and higher PVAs tend to have better optical quality, and the determination of the fundamentals and definitions of the parameters have been described previously (16).

### Corneal aberrations measurement

The total corneal aberrations in a 6-mm zone were obtained from corneal tomography. Parameters of corneal aberrations included total RMS, LOA RMS (referring to the Zernike coefficients of order 2), and HOA RMS (referring to the Zernike coefficients of order greater than 2). In general, participates with lower RMS tend to have better optical quality.

### Quality of life impact of refractive correction

Quality of Life Impact of Refractive Correction (QIRC) questionnaire was developed and validated for assessing the quality of life of people with refraction corrections, including those who accept refractive surgery (17, 18). A total of 20 items are included in this scale under the following four modules: postoperative symptoms, visual and physical functions, social activities, and mental health. In general, higher scores represent subjects with higher visual quality for life.

### Statistical analysis

All statistical analyses were performed using SPSS version 24.0 (SPSS Inc., IBM, Unied States). The results are expressed as the mean ± SD. A normal distribution was determined using the Kolmogorov–Smirnov test. Use a paired *t*-test on data that fits a normal distribution. Comparison of data between multiple groups using analysis of variance (ANOVA) with Bonferroni. A *p* value less than 0.05 was considered statistically significant.

## Results

**Figure 1:**
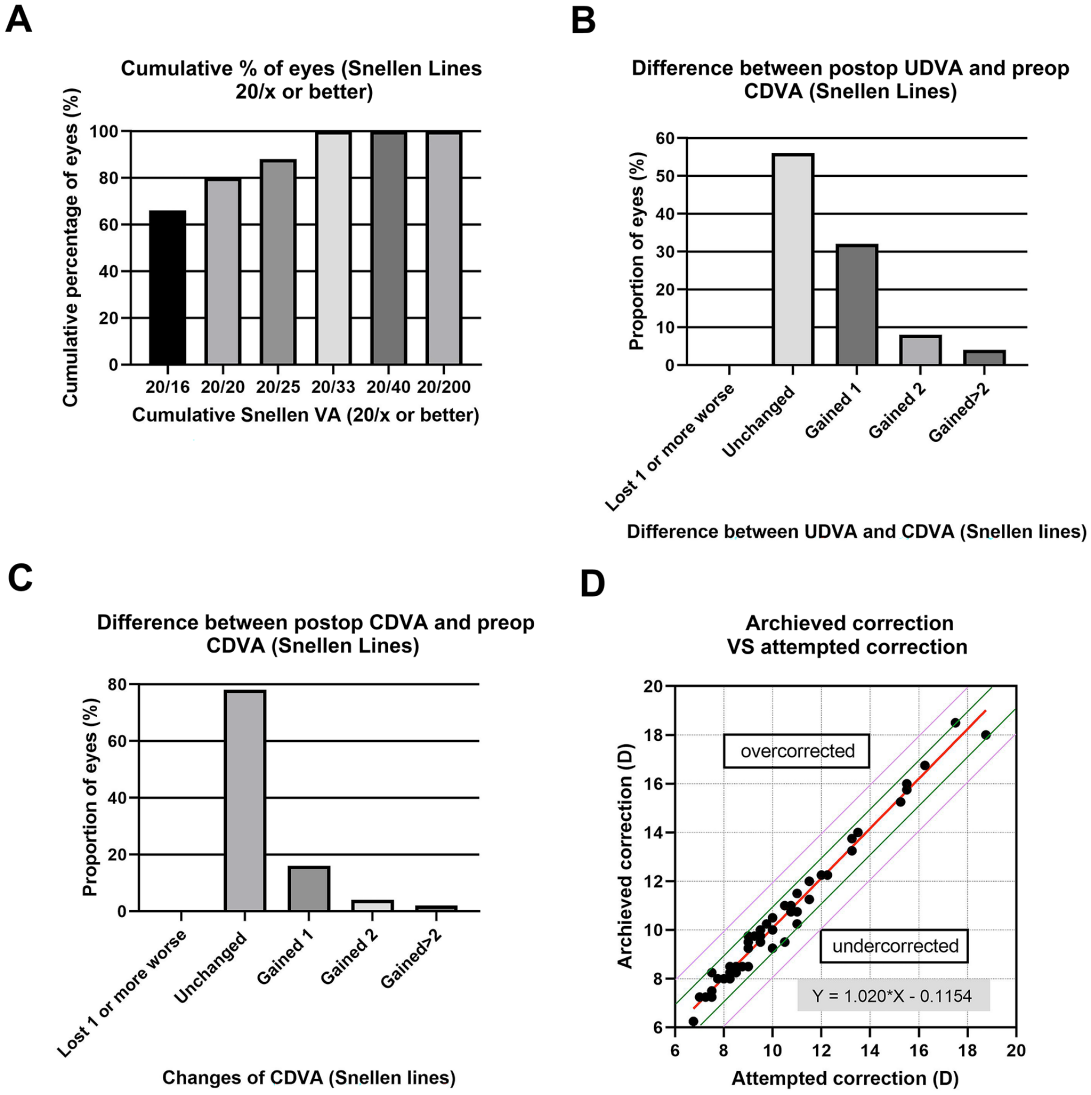
Refractive outcomes at 2 weeks postoperatively after implantation of ICL V4c, including **(A)** Cumulative percentage of eyes attaining specified levels of uncorrected distance visual acuity (UDVA); **(B)** Postoperative vs. preoperative UDVA; **(C)** Change in corrected distance visual acuity (CDVA); and **(D)** Target-induced astigmatism plotted vs. surgical-induced astigmatism at the last follow-up.

The study comprised 80 patients. No intraoperative or short-term postoperative events were observed in any of the eyes. At 2 weeks postoperatively, the mean logMAR UDVA and CDVA were − 0.01 ± 0.12 and − 0.08 ± 0.12, respectively. The mean values of the efficacy indexes of all 80 eyes were 1.18 ± 0.19 (Figure 1A), all the eyes had postoperative UDVA ≥20/33, and 82% of the eyes achieved better than 20/20. None of the eyes lost 1 or more lines of CDVA, 16% remained unchanged, 78% gained 1 line, and 4% gained 2, and 2% gained more than 2 lines (Figure 1B). The mean values of the safety indexes of all 80 eyes were 1.23 ± 0.21. In 56% of the eyes (Figure 1C), the preoperative CDVA was maintained, in 32%, it increased by one line, in 8%, it increased by two lines, in 4% it by more than two lines, and no CDVA declined. A scatterplot and the best linear fit line (*r* = 0.9776) of the attempted versus the achieved SE correction are shown in Figure 1D. Of all the eyes, 94% were within ±1.00 D (Figure 1D, green lines) and 100% were within ±2.00 D of the desired SE refraction (Figure 1D, purple lines).

### Pupillary characteristics

As shown in Table 1, 0.2 and 0.15% groups had no significant difference in the static and dynamic pupillary parameters before instillation (*p* > 0.05). Pupil diameter prior to installation of the 0.2 and 0.15% group had no significant differences (*p* > 0.05). After instillation, the scotopic PD, mesopic PD and photopic PD of the 0.2% post and the 0.15% post were both significantly smaller than pre-PD. The pupil dilation velocities within 1S, 2S, and 1S-2S after LS of both groups were significantly slower than those before instillation. While there was no statistical difference in the static and dynamic PD and velocity data after LS between the two groups.

**Table 1:** Pupil size and pupil dynamics in brimonidine-treated eyes before, and at 1/2 h after instillation.

	0.2% Pre	0.15% Pre	0.2% Post	0.15% Post	*p*^a^ value	*p*^b^ value	*p* ^c^ value	*p* ^d^ value
Scotopic PD (mm)	6.58 ± 0.75	6.47 ± 0.76	5.16 ± 1.15	4.88 ± 1.09	0.859	0.209	**<0.001**	**0.008**
Mesopic PD (mm)	5.04 ± 0.94	4.96 ± 0.77	4.06 ± 0.89	3.95 ± 0.63	0.653	0.384	**0.001**	**0.001**
Photopic PD (mm)	4.08 ± 0.77	4.20 ± 0.54	3.48 ± 0.47	3.35 ± 0.62	0.398	0.083	**0.002**	**<0.001**
Initial PD (mm)	4.07 ± 0.64	4.11 ± 0.69	3.17 ± 0.42	3.08 ± 0.37	0.305	0.406	**0.008**	**0.003**
Minimum PD after LS (mm)	3.73 ± 0.64	3.53 ± 0.48	3.23 ± 0.50	3.18 ± 0.53	0.166	0.503	**0.006**	**0.027**
V_0–1 s_ after LS (mm/s)	0.73 ± 0.15	0.76 ± 0.21	0.41 ± 0.13	0.50 ± 0.20	0.582	0.059	**<0.001**	**<0.001**
V_0–2 s_ after LS (mm/s)	0.58 ± 0.11	0.64 ± 0.16	0.29 ± 0.10	0.33 ± 0.14	0.221	0.340	**<0.001**	**0.001**
V_1–2 s_ after LS (mm/s)	0.43 ± 0.11	0.51 ± 0.15	0.17 ± 0.12	0.16 ± 0.13	0.171	0.859	**<0.001**	**<0.001**

Pre, before instillation of brimonidine; Post, 30 min after instillation of brimonidine.

*p*^a^ values for the analysis of *t*-tests performed between 0.2% Pre and 0.15% Pre.

*p*^b^ values for the analysis of *t*-tests performed between 0.2% Post and 0.15% Post.

*p*^c^ values for the analysis of *t*-tests performed between 0.2% Pre and 0.2% Post.

*p*^d^ values for the analysis of *t*-tests performed between 0.15% Pre and 0.15% Post.

### Pupil center

**Figure 2:**
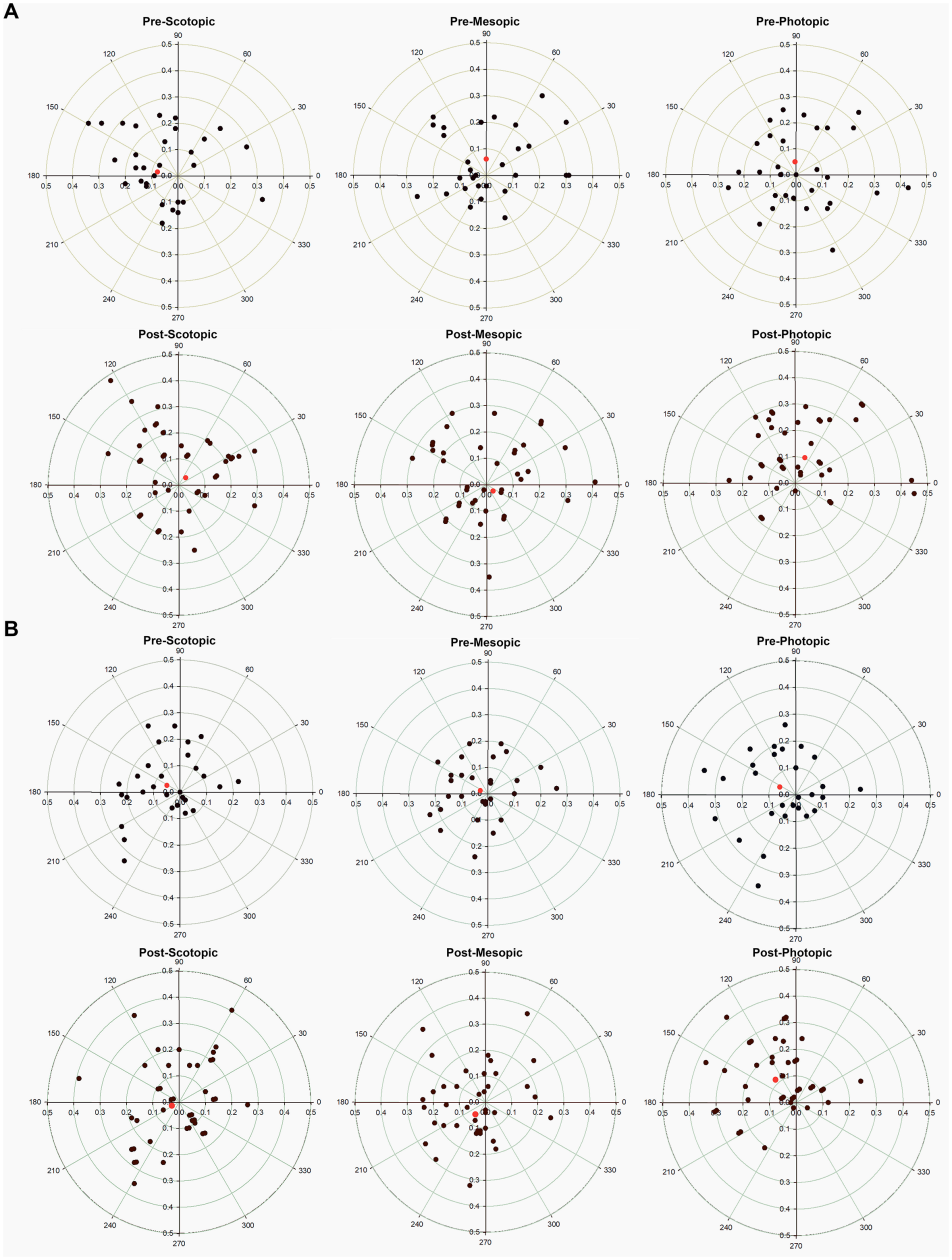
Scatter plot showing the pupil center (colored data points) in comparison to the corneal vertex (geographic center) pre-and post-treatment in different light environment for the **(A)** 0.2% group, and **(B)** 0.15% group. The rings represent 0.1-mm intervals. The red diamond shows the vector mean of the population.

Pupil center coordinates prior to installation of two groups had no significant differences (Table 2, *p* > 0.05). 0.2% group had significant changes in pupil center before and after treatment. Among them, the *X*-axis coordinates of the scotopic pupil center in the 0.2% group moved from−0.08 ± 0.14 to 0.03 ± 0.17 mm (*p* = 0.006), showing that the pupil center moved to the nasal side (Figure 2A). The *Y*-axis coordinate of the mesopic pupil center in the 0.2% group moved from 0.06 ± 0.11 to −0.03 ± 0.50 mm (*p* = 0.046), showing the pupil center moved downward (Figure 2B). While in the photopic environment, the *X*-axis and *Y*-axis of the pupil centers did not change significantly. There was no obvious movement of the pupil center of the 0.15% group compared with that before administration under all illumination condition (*p* > 0.05).

**Table 2:** Mean pupil barycentre configurations in brimonidine-treated eyes before, and at 1/2 h after instillation.

		0.2% Pre	0.15% Pre	0.2% Post	0.15% Post	*p*^a^ value	*p*^b^ value	*p*^c^ value	*p*^d^ value
Scotopic	Xc (mm)	−0.08 ± 0.14	−0.05 ± 0.12	0.03 ± 0.17	−0.03 ± 0.17	0.630	0.478	**0.006**	0.218
	Yc (mm)	0.04 ± 0.12	0.02 ± 0.13	0.03 ± 0.46	−0.01 ± 0.55	0.246	0.848	0.813	0.754
Mesopic	Xc (mm)	0.00 ± 0.14	−0.03 ± 0.12	0.02 ± 0.18	−0.04 ± 0.14	0.604	0.515	0.946	0.933
	Yc (mm)	0.06 ± 0.11	0.01 ± 0.10	−0.03 ± 0.50	−0.06 ± 0.52	0.229	0.913	**0.046**	0.584
Photopic	Xc (mm)	0.00 ± 0.16	−0.06 ± 0.14	0.03 ± 0.17	−0.08 ± 0.11	0.249	0.140	0.811	0.952
	Yc (mm)	0.04 ± 0.12	0.03 ± 0.13	0.10 ± 0.11	0.09 ± 0.10	0.618	0.830	0.071	0.076

Pre, before instillation of brimonidine; Post, 30 min after instillation of brimonidine.

*p*^a^ values for the analysis of *t*-tests performed between 0.2% Pre and 0.15% Pre.

*p*^b^ values for the analysis of *t*-tests performed between 0.2% Post and 0.15% Post.

*p*^c^ values for the analysis of *t*-tests performed between 0.2% Pre and 0.2% Post.

*p*^d^ values for the analysis of *t*-tests performed between 0.15% Pre and 0.15% Post.

### Visual quality

Table 3 shows the pre-instillation and post-instillation optical quality. Both in the 0.2 and 0.15% groups, all optical quality parameters were significantly improved after treatment with brimonidine eye drops. The bright MTF after treatment of the 0.15% group was higher than that of 0.2% group (*p* = 0.004). And the dark OSI after treatment of the 0.15% group was lower than that of 0.2% group (*p* = 0.012).

**Table 3:** Optical quality parameters in brimonidine-treated eyes before and at 1/2 h after instillation under different lighting conditions.

		0.2% Pre	0.15% Pre	0.2% Post	0.15% Post	*p*^a^ value	*p*^b^ value	*p*^c^ value	*p*^d^ value
Bright	OSI	1.09 ± 0.53	1.10 ± 0.50	0.86 ± 0.56	0.82 ± 0.55	0.928	0.375	**0.034**	**0.036**
	MTF	35.44 ± 0.55	36.05 ± 0.53	41.01 ± 0.56	44.07 ± 0.54	0.915	**0.004**	**0.028**	**0.017**
	SR	0.21 ± 0.07	0.21 ± 0.69	0.23 ± 0.12	0.23 ± 0.16	0.695	0.116	**0.031**	**0.003**
	PVA100%	1.13 ± 0.07	1.14 ± 0.09	1.32 ± 0.08	1.33 ± 0.09	0.778	0.229	**0.019**	**0.038**
	PVA20%	0.87 ± 0.03	0.86 ± 0.04	0.99 ± 0.07	1.00 ± 0.06	0.375	0.441	**0.021**	**0.037**
	PVA9%	0.53 ± 0.04	0.54 ± 0.02	0.58 ± 0.02	0.61 ± 0.03	0.562	0.117	**0.047**	**0.039**
Dark	OSI	1.05 ± 0.35	1.03 ± 0.41	0.89 ± 0.49	0.78 ± 0.40	0.834	**0.012**	**0.032**	**0.018**
	MTF	26.95 ± 0.49	27.01 ± 0.51	31.56 ± 0.48	34.71 ± 0.58	0.732	0.142	**0.021**	**0.002**
	SR	0.15 ± 0.03	0.16 ± 0.02	0.18 ± 0.03	0.19 ± 0.03	0.683	0.384	**0.003**	**0.032**
	PVA100%	0.90 ± 0.06	0.89 ± 0.07	1.10 ± 0.06	1.13 ± 0.07	0.372	0.102	**0.006**	**0.023**
	PVA20%	0.62 ± 0.05	0.60 ± 0.04	0.79 ± 0.03	0.80 ± 0.04	0.424	0.310	**0.003**	**0.002**
	PVA9%	0.37 ± 0.04	0.38 ± 0.03	0.44 ± 0.04	0.48 ± 0.03	0.243	0.090	**0.027**	**0.041**

Pre, before instillation of brimonidine; Post, 30 min after instillation of brimonidine.

*p*^a^ values for the analysis of *t*-tests performed between 0.2% Pre and 0.15% Pre.

*p*^b^ values for the analysis of *t*-tests performed between 0.2% Post and 0.15% Post.

*p*^c^ values for the analysis of *t*-tests performed between 0.2% Pre and 0.2% Post.

*p*^d^ values for the analysis of *t*-tests performed between 0.15% Pre and 0.15% Post.

### Corneal aberrations and QIRC score

Table 4 shows the pre-instillation and post-instillation scotopic corneal aberrations. Both in the 0.2 and 0.15% groups, all corneal aberrations parameters were significantly improved after treatment with brimonidine eye drops (shown as LOA RMS, HOA RMS, RMS, and SR). The HOA RMS of the 0.15% group decreased from 0.27 ± 0.69 to 0.21 ± 0.16 (*p* = 0.003), while the HOA RMS of the 0.2% group had no significant differences before and after treatment. The QIRC score of the 0.2 and 0.15% groups was significantly higher than that of the point before treatment. The QIRC score in the 0.15% group after treatment was significantly higher than that in the 0.2% group (*p* = 0.043).

**Table 4:** Scotopic corneal aberrations in brimonidine-treated eyes before, and at 1/2 h after instillation.

	0.2% Pre	0.15% Pre	0.2% Post	0.15% Post	*p*^a^ value	*p*^b^ value	*p*^c^ value	*p*^d^ value
RMS	1.09 ± 0.53	1.10 ± 0.50	1.06 ± 0.56	1.02 ± 0.55	0.928	0.375	**0.034**	**0.036**
LOA RMS	1.04 ± 0.55	1.05 ± 0.53	1.01 ± 0.56	1.07 ± 0.54	0.915	0.054	**0.028**	**0.010**
HOA RMS	0.27 ± 0.07	0.27 ± 0.69	0.28 ± 0.12	0.21 ± 0.16	0.695	**0.016**	0.531	**0.003**
QIRC score	43.89 ± 5.17	44.10 ± 4.98	46.63 ± 6.03	47.33 ± 4.83	0.703	**0.043**	**0.017**	**0.012**

Pre, before instillation of brimonidine; Post, 30 min after instillation of brimonidine.

*p*^a^ values for the analysis of *t*-tests performed between 0.2% Pre and 0.15% Pre.

*p*^b^ values for the analysis of *t*-tests performed between 0.2% Post and 0.15% Post.

*p*^c^ values for the analysis of *t-*tests performed between 0.2% Pre and 0.2% Post.

*p*^d^ values for the analysis of *t*-tests performed between 0.15% Pre and 0.15% Post.

### Manifest refraction and IOP

As showed in Table 5, the spherical refraction, cylindrical refraction, and equivalent spherical of 0.2 and 0.15% group before and after installation had no significant differences (*p* > 0.05). Both 0.2 and 0.15% post-installation showed a significant IOP decreases (*p* = 0.015, *p* = 0.034), while there was no statistically significant difference between post-0.2% and post-0.15% (*p* > 0.05). Both concentrations of brimonidine eye drops can steadily reduce intraocular pressure without changing spherical refraction, cylindrical refraction, and equivalent spherical.

**Table 5:** Refraction parameters and IOP in brimonidine-treated eyes before, and at 1/2 h after instillation.

	0.2% Pre	0.15% Pre	0.2% Post	0.15% Post	*p*^a^ value	*p*^b^ value	*p*^c^ value	*p*^d^ value
Spherical refraction (D)	0.39 ± 0.41	0.21 ± 0.47	0.40 ± 0.55	0.37 ± 0.64	0.156	0.832	0.855	0.617
Cylindrical refraction (D)	−0.50 ± 0.35	−0.25 ± 0.36	−0.51 ± 0.23	−0.35 ± 0.60	0.229	0.426	0.812	0.645
Spherical equivalent (D)	0.13 ± 0.41	0.10 ± 0.50	0.20 ± 0.51	0.19 ± 0.74	0.974	0.961	0.581	0.802
IOP (mmHg)	15.67 ± 1.28	14.65 ± 2.04	12.38 ± 1.88	11.12 ± 2.11	0.282	0.704	**0.015**	**0.034**

Pre, before instillation of brimonidine; Post, 30 min after instillation of brimonidine.

*p*^a^ values for the analysis of *t*-tests performed between 0.2% Pre and 0.15% Pre.

*p*^b^ values for the analysis of *t*-tests performed between 0.2% Post and 0.15% Post.

*p*^c^ values for the analysis of *t*-tests performed between 0.2% Pre and 0.2% Post.

*p*^d^ values for the analysis of *t*-tests performed between 0.15% Pre and 0.15% Post.

## Discussion

Clinically, a few patients complain of apparent ring-shaped dysphotopsia in the early postoperative period after ICL V4c implantation (11). Brimonidine tartrate 0.2 and 0.15% ophthalmic solution has been shown to improve the symptoms of ring-shaped dysphotopsia in postoperative ICL patients *via* pharmacologic miosis (14, 15). But no literature exists reporting the differences in the anti-mydriatic effects of the two concentrations. Therefore, this study is the first to compare the anti-mydriatic effect, visual quality, and effect on the pupil center of brimonidine 0.2 and 0.15% in different brightness environments.

Various concentrations of brimonidine (0.1, 0.15, and 0.2%) demonstrated their anti-mydriatic effect, which can be used to reduce night vision problems such as halos and glare after laser refractive surgery (14, 15, 19, 20). The anti-mydriatic effect of brimonidine on pupil diameter results from brimonidine’s a2 agonist activity, which reduces the production, storage and release of norepinephrine into synapses, and thus inhibiting iris dilation (13–15, 19–21). Although the anti-mydriatic effect of brimonidine has been shown in scotopic, mesopic, and photopic conditions, previous research suggested that the anti-mydriatic effect was more prominent in scotopic states because norepinephrine is the primary mediator of nocturnal pupil dilation, brimonidine inhibits the amount of pupil dilation at night by reducing the release of norepinephrine (19–21). In addition, since brimonidine does not induce a miotic effect, the amount of reduction in pupil size is limited (15, 19).

Similar to the previous study, we found that pupil size was significantly decreased under scotopic, mesopic and photopic conditions 30 min after brimonidine tartrate 0.15 and 0.2% instillation. Scotopic PD reduced from 6.58 ± 0.75 to 5.16 ± 1.15 mm after brimonidine tartrate 0.2%, while scotopic PD after brimonidine tartrate 0.15% decreased from 6.47 ± 0.76 to 4.88 ± 1.09 mm. Thorsen et al. reported that under scotopic conditions, 100 and 60% of healthy eyes had a reduction in pupil size of ≥1.0 mm at 30 min and 6 h, respectively (22). The results of previous studies showed that the scotopic PD minimized from 7.2 ± 0.4 to 5.5 ± 0.8 mm after 0.2% brimonidine instillation (15); the mean pupil size reduced from 6.09 ± 1.03 to 4.45 ± 1.04 mm after 0.15% brimonidine instillation (14); the photopic and scotopic PD before 0.15% brimonidine instillation was 4.8 ± 1.2 and 5.8 ± 1.2 mm, respectively, and 30 min after instillation, the photopic and scotopic PD decreased to 4.3 ± 1.1 and 5.3 ± 1.0 mm, respectively (23). The present study showed that brimonidine 0.15 and 0.2% had the same anti-mydriatic effect under scotopic, mesopic, and photopic conditions, as well as initial PD and Minimum PD after LS is basically consistent with previous research data trends.

The speed of pupil dilation after light stimulation of 0.2 and 0.15% post-instillation was significantly slower than pre-instillation, and there was no statistical difference between the 0.2 and 0.15% groups. Recent studies had the similar trend, compared with pre-instillation measurements, 0.15% brimonidine treated eyes had significantly lower pupil dilation velocity in all studied seconds (23).

In the present study, the pupil center tended to be closer to the corneal center (the geographic center of the radar map) after treatment with two concentrations of brimonidine eye drops. Especially in scotopic and medium scotopic vision, the data of the 0.15% group are more statistically significant. The pupil center plays a central role in the optical system of the eye. Retinal image quality is significantly affected by the center of the pupil and the center of the lens. Postoperative pupillary center displacement is considered for two reasons: Firstly, pupils constrict due to the anti-mydriatic effect of brimonidine, which conduct to slight displacement of pupil center. Studies have found a correlation between pupillary excursion (the distance from the center of the pupil to the center of the cornea) and dark pupil diameter, which increases pupillary excursion when pupil diameter is increased by changing lighting conditions from photopic to mesopic, the displacement increased with the pupil diameter of the subjects (24, 25). The second reason is that vault changes with the pupil, which has certain mechanical stimulation to the iris tissue, resulting in subtle changes in the pupil center. Moreover, the latest research confirmed that brimonidine induce reduction of blood circulation in the ciliary body and iris (26), which might cause slight changes in iris movement.

The spherical refraction, cylindrical refraction, and spherical equivalent before and after instillation had no significant changes in both groups. The reduction of intraocular pressure had no statistical differences between two groups, which is consistent with the results of previous studies. Previous studies have indicated that brimonidine tartrate reduces intraocular pressure by reducing aqueous humor production and increasing uveoscleral output, and has demonstrated the efficacy and safety of brimonidine in reducing intraocular pressure (27). Numerous studies suggest that preservatives may cause severe ocular toxicity or reduced tolerance, especially after prolonged chronic use and/or frequent dosing, and are often associated with increased OSD frequency. As mentioned earlier, topical antiglaucoma treatments may cause local side effects due to their active ingredients, preservatives, or excipients. Previous studies have shown significant negative changes in tear parameters after 4 weeks of use with preservative brimonidine eye drops. The previous study also compared the efficacy and safety of brimonidine 0.15% twice daily with brimonidine 0.2% twice daily in POAG or OHT patients and concluded that in POAG or OHT patients, brimonidine Nitidine 0.15% provided comparable IOP reduction to brimonidine 0.2%. The advantage of reducing side effects (e.g., allergic conjunctivitis) increased patient satisfaction (28).

The data of this study showed that with 0.15 and 0.2% concentration of brimonidine eye drops after half an hour of treatment, the visual quality in both bright and dark environments had significantly improved compared with that before the treatment. Among them, the 0.15% group had significantly higher visual quality than the 0.2% group in the dark environment, manifested in higher OSI and HOA RMS. the 0.15% group also had higher MTF values than the 0.2% group in the bright environment. There could be two explanations for these results. Firstly, 0.15% brimonidine has a better corneal microenvironment after instillation because it is free of preservatives (28). Secondly, the 0.15% group had minor HOA RMS in the dark environment than the 0.2% group, which is consistent with the recent research. The corneal higher-order aberrations dependent on corneal microvilli correlated significantly with haloes and optical quality (29). Moreover，recent research has proven the halo size correlated independently with OSI (30–32).

There are two limitations of this study. Firstly, it requires more time points after surgery or the instillation of brimonidine tartrate eye drops for further detection and observation of the anti-mydriatic effect. Our team will conduct further research and verification in follow-up experiments. The second is the relatively small sample size, more healthy subjects are needed to confirm our initial findings.

In conclusion, both 0.15 and 0.2% concentrations of brimonidine tartrate eye drops have been shown to be effective postoperative treatment options for improving night vision quality after ICL V4c implantation. This study showed that 0.15 and 0.2% brimonidine tartrate eye drops had similar anti-mydriatic ability, while 0.15% group had better visual quality than 0.2% concentration, and hardly introduced pupil shift. In conclusion, 0.15% brimonidine tartrate eye drops may be more suitable for patients with nocturnal glare symptoms in the early postoperative period after ICL implantation.

## Data availability statement

The raw data supporting the conclusions of this article will be made available by the authors, without undue reservation.

## Ethics statement

The studies involving human participants were reviewed and approved by the Clinical Trial Management System of the First Affiliated Hospital of Zhengzhou University (2021-KY-0872-002). The patients/participants provided their written informed consent to participate in this study.

## Author contributions

JY implemented the study, collected the data, analyzed the data, and wrote the article. JY, WW, and MZ participated in the design and interpretation of the studies, analysis of the data, and review of the article. All authors contributed to the article and approved the submitted version.

## Funding

This work was supported by the Henan Provincial Medical Science and Technology Project Jointly Established Project (LHGJ20200337).

## Conflict of interest

The authors declare that the research was conducted in the absence of any commercial or financial relationships that could be construed as a potential conflict of interest.

## Publisher’s note

All claims expressed in this article are solely those of the authors and do not necessarily represent those of their affiliated organizations, or those of the publisher, the editors and the reviewers. Any product that may be evaluated in this article, or claim that may be made by its manufacturer, is not guaranteed or endorsed by the publisher.

## Footnotes

^1^ https://evo-ocos.staarag.ch
