# Supplementary material for: Effects of brimonidine tartrate 0.2 and 0.15% ophthalmic solution on the static and dynamic pupil characteristics
Source: Front Med (Lausanne). 2023 May 15;10:1160414. doi: 10.3389/fmed.2023.1160414 (PMC10225522; doi:10.3389/fmed.2023.1160414)
Supplement: Supplementary file 1 [file Data_Sheet_1.docx]

**Supplementary File S1. Demographic data of the study population pre-/ post- V4c ICL implantation**

| **Parameter** | | | | |
| --- | --- | --- | --- | --- |
| Number of patients (eyes) | | 80 （80） | | |
| Sex (male: female) | | 37：43 | | |
| Age (years) | | 25.35±4.59 | | |
| WTW (mm) | | 11.54±0.35 | | |
| ACD (mm) | | 3.15±0.24 | | |
| CCT (μm) | | 517.92±27.41 | | |
| ICL Size (mm) | | 12.69±0.39 | | |
|  | **Preoperative** | | **Postoperative** | **P Value** |
| UDVA (logMAR) | 1.42±0.42 | | 0±0.11 | <0.003 |
| CDVA (logMAR) | 0.07±0.11 | | -0.08±0.12 | <0.001 |
| Spherical (D) | -9.07±3.17 | | 0.26±0.49 | <0.001 |
| Cylindrical (D) | -1.64±1.06 | | -0.38±0.37 | <0.006 |
| Spherical equivalent (D) | -9.92±3.38 | | 0.10±0.45 | <0.001 |
| Axial length (mm) | 27.25±1.26 | | 27.24±1.30 | 0.638 |
| ECD (cells/mm^2^) | 2899.19±216.93 | | 2735.32±201.47 | 0.199 |
| IOP (mmHg) | 16.36±2.41 | | 15.48±1.71 | 0.989 |
