# Supplementary figures and images for: Effects of brimonidine tartrate 0.2 and 0.15% ophthalmic solution on the static and dynamic pupil characteristics
Source: Front Med (Lausanne). 2023 May 15;10:1160414. doi: 10.3389/fmed.2023.1160414 (PMC10225522; doi:10.3389/fmed.2023.1160414)

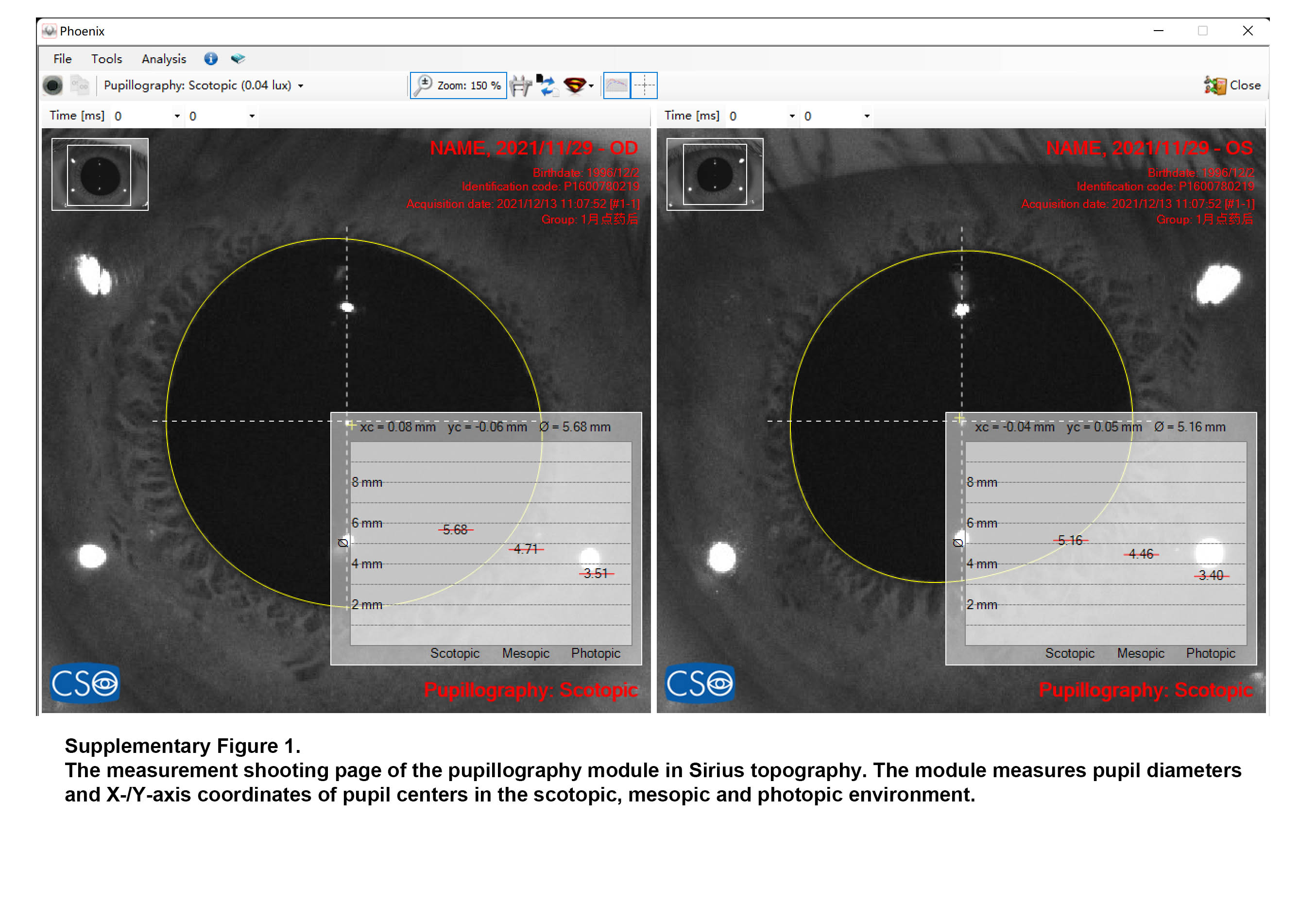

Supplement: Supplementary file 2 [file Image_1.jpg]

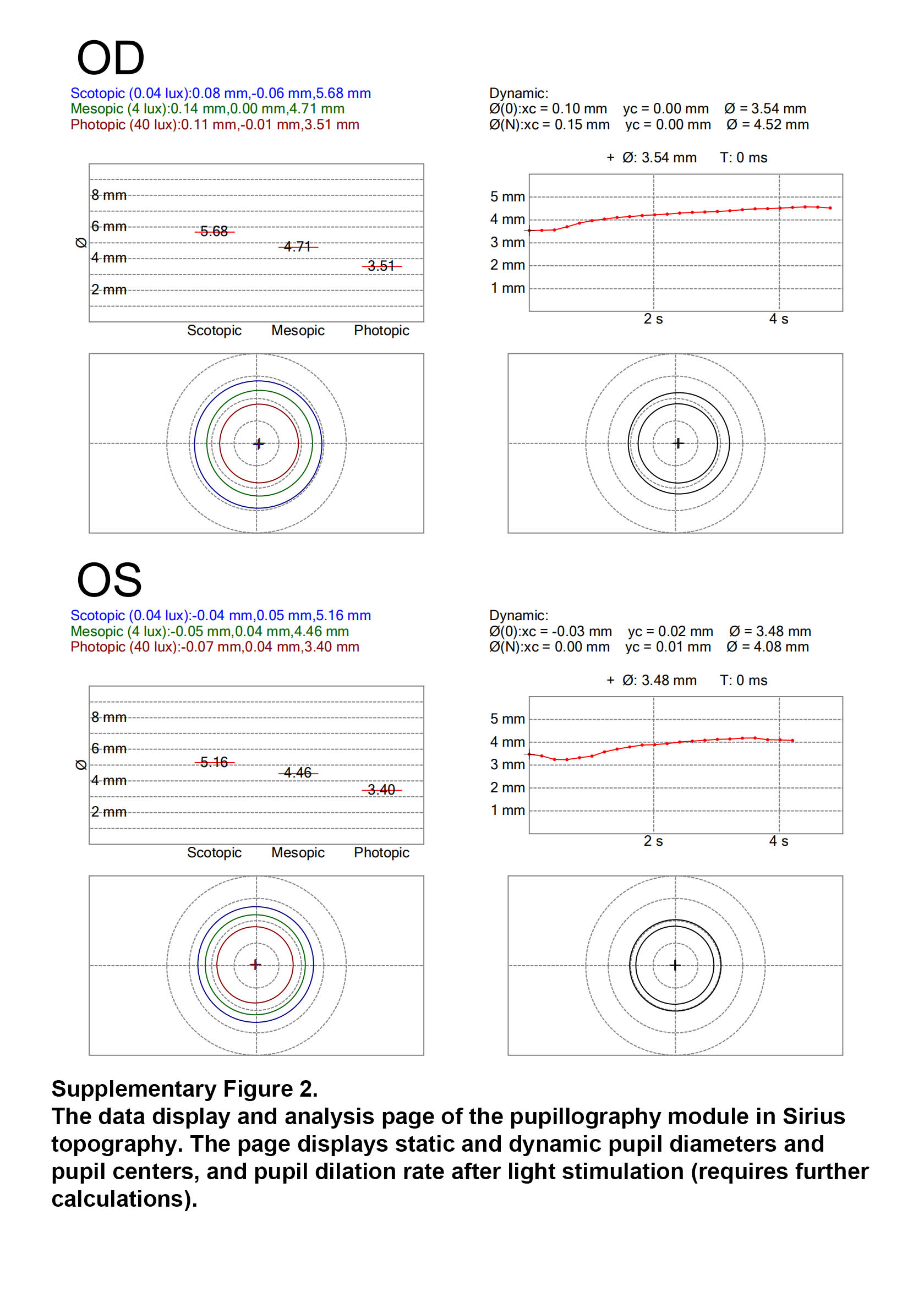

Supplement: Supplementary file 3 [file Image_2.jpg]
